# The role of confocal laser endomicroscopy in pediatric gastrointestinal diseases: a narrative review

**DOI:** 10.3389/fped.2025.1681649

**Published:** 2025-09-29

**Authors:** Irene Dalpiaz, Luca Scarallo, Marco Andreini, Sara Renzo, Giusy Russo, Cosimo Ruggiero, Danila Volpe, Paolo Lionetti, Salvatore Oliva

**Affiliations:** ^1^ Gastroenterology and Nutrition Unit, Meyer Children’s Hospital IRCCS, Florence, Italy; ^2^ Department NEUROFARBA, University of Florence, Florence, Italy; ^3^ Pediatric Gastroenterology and Liver Unit, Department of Maternal Infantile and Urological Sciences, Sapienza University of Rome, Rome, Italy

**Keywords:** confocal laser endomicroscopy, pediatric gastrointestinal diseases, inflammatory bowel diseases, food allergy, functional gastrointestinal disorders

## Abstract

**Introduction:**

Confocal laser endomicroscopy (CLE) is an innovative tool that enables microscopic evaluation of the gastrointestinal mucosa during the digestive endoscopy, providing real-time diagnostic information alongside histopathological findings.

**Methods:**

A literature search on CLE in pediatric gastroenterology was performed.

**Results:**

CLE has a broad range of applications, spanning from the upper digestive tract to the lower digestive tract, but its applications in the pediatric setting remains largely unexplored and confined to experimental setting.

**Discussion:**

Despite the extensive potential of CLE, its application in pediatric patients has been poorly investigated. This narrative review aims to consolidate the current knowledge on CLE in gastrointestinal diseases and to draw insights from adult studies to promote future research in the pediatric field.

## 1 Introduction

Endoscopy plays a pivotal role in the diagnosis and management of pediatric gastrointestinal disease. Since its introduction in the 1970s, the field has undergone significant advancements driven by computer processing and imaging technologies, such as the microscopic tissue characterization, exemplified by confocal laser endomicroscopy (CLE).

CLE, distinguished by its ability to produce high-magnification and high-resolution images, represents a promising advancement in endoscopic technology. Its primary strength lies in enabling real-time, *in vivo*, histological tissue analysis, providing information on microarchitecture and physiological mechanisms (1). Consequently, by integrating the endoscopic evaluation with the histological one, CLE may reduce times for diagnosis and subsequent management (2).

While substantial evidence supports the use of CLE in adults, spanning gastrointestinal and biliopancreatic pathology, particularly gastrointestinal neoplasia and inflammatory bowel diseases IBD), its application in pediatric patients remains largely unexplored (3).

This narrative review aims to consolidate the current knowledge of CLE in pediatric gastrointestinal diseases. Furthermore, it seeks to extrapolate potential applications based on insights derived from adult studies, thereby contributing to the evolving landscape of pediatric endoscopy.

## 2 Methods

We performed a literature search in Medline through PubMed using keywords related to CLE in pediatric gastroenterology: “confocal laser endomicroscopy” or “endomicroscopy” or “CLE” and “children” or “pediatric” or “paediatric” and “gastrointestinal” or “gastric” or “esophageal” or “digestive” or “inflammatory bowel diseases” or “Crohn's disease” or “ulcerative colitis” or “celiac disease” or “gastro-esophageal reflux disease” or “eosinophilic esophagitis” or “irritable bowel syndrome” or “functional gastrointestinal disorders”. Original studies and review articles were identified up to December 2024. Studies that were not published in English were excluded. References of selected articles were included if pertinent.

Original studies that were conducted in children or both in children and adults and included as result of the search are summarized in Table 1.

**Table 1:** Original studies on confocal laser endomicroscopy in pediatric gastrointestinal diseases.

Author (year)	Study design	Country	Sample size	Median age (IQR^a^)	Main findings	Limits
Venkatesh et al. 2012 (20)	Retrospective observational study	UK	23 children (7 patients and 16 controls)	Patients: 7.6 y^b^ (1.8−15.5 y) Controls: 12 y (2.2–15.3 y)	Higher surface to papillary distance in controls: measurement of the distance enabling real-time diagnosis of GERD^c^	Study design (single-centered study); small sample size; reliance on only papillary elongation in arriving at a diagnosis
Neumann et al. 2011 (21)	Case report	Germany	1 adolescent	18 y	Anomalies associated with EoE^d^: dilated intercellular spaces, capillary ectasia within the esophageal squamous epithelium, fluoresceine leakage	Study design (case report)
Yoo et al. 2011 (22)	Prospective ex vivo observational study	USA	43 biopsy samples (35 from children, 8 from adults)	Children: 11.2 y (1.8–21.1 y) Adults: not specified	Accuracy of CLE in intraepithelial eosinophils count and identification of microscopic anomalies associated with EoE	Imaging not conducted *in vivo*
Venkatesh et al. 2010 (28)	Prospective observational study	Australia	19 children (9 patients, 10 matched controls)	Patients: 8.35 y (2–12.66 y)	High sensitivity, specificity, and positive predictive value for CLE in comparison to the histology (100%, 80%, 81%, respectively)	Study design (single-centered study); small sample size
Shavrov et al. 2016 (34)	Prospective observational study	Russian Federation	24 children and adolescents (13 Crohn's disease, 11 ulcerative colitis)	14 y (10–21)	Increased epithelial gap density in the terminal ileum predictive of disease relapse (*p*-value 0.02)	Study design (single-centered study); small sample size
Shimojima et al. 2020 (46)	Preclinical ex vivo observational study	Japan	69 colon samples from 9 children who underwent surgery	Not specified	Observation of the enteric nervous system in the ganglionic segment but not in the aganglionic one by CLE	Study design (single-centered study); imaging not conducted *in vivo*
Harada et al. 2021 (47)	Case series	Japan	2 children	Patient 1: 2 y Patient 2: 1 y, 5 months	Potential of intra-operatively enteric nervous system identification by using CLE during surgery	Study design (case series)

^a^ IQR, interquartile range; ^b^y, years; ^c^GERD, gastroesophageal reflux disease; ^d^EoE, eosinophilic esophagitis.

## 3 Confocal laser endomicroscopy technology

**Figure 1:**
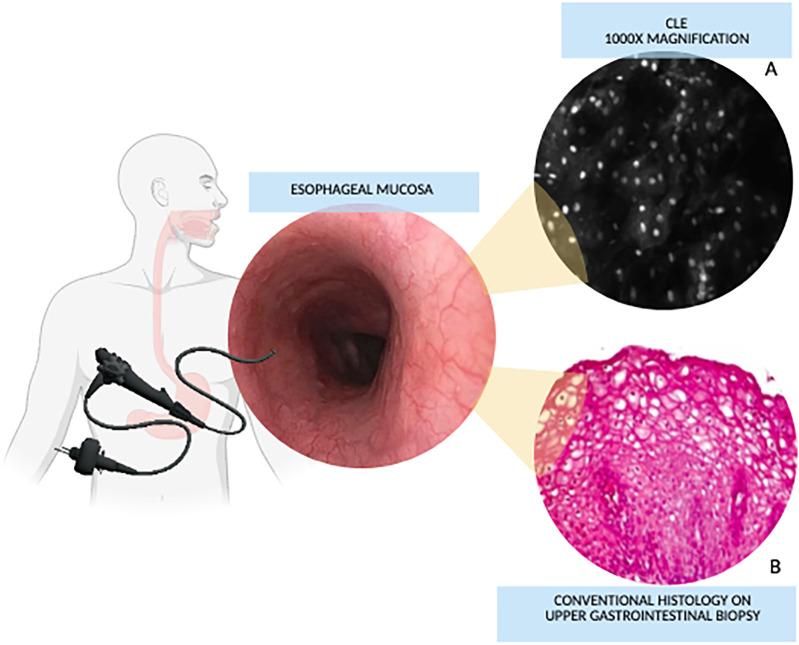
Confocal Laser endomicroscopy vs. conventional histology magnification. **(A)** Confocal image of non-keratinized squamous epithelium of the esophagus. **(B)** Histological image of esophagus. Images from: Venkatesh K, et al. Feasibility of confocal endomicroscopy in the diagnosis of pediatric gastrointestinal disorders. *World J Gastroenterol 2009*; 15:2214–2219. doi: 10.3748/wjg.15.2214.

CLE represents a significant advancement over conventional endoscopy, which relies on white light illumination. CLE employs low-power laser illumination focused on a single point of interest, enabling microscopic field image acquisition. Images are generated by collecting light reflected from tissue through a pinhole and directing it to a detector, which converts the signal into grayscale images. The term “confocal” refers to the alignment of the illumination and detection systems within the same focal plane, significantly enhancing image clarity (4). CLE operates a scan rate of 1.6 frames or 0.8 frames per second, with a scanning depth ranging from 0 to 250 μm, enabling comprehensive exploration of tissue layers. Its 1000-fold magnification facilitates the detailed visualization of microscopic structures (3, 4). Figure 1 shows the high grade of magnification obtained by CLE compared to the conventional histology on biopsies.

Two CLE systems have been developed: endoscope-based CLE (eCLE) and probe-based CLE (pCLE). Currently, only pCLE is available for clinical use. eCLE integrates a confocal device directly into an endoscope, whereas pCLE employs a probe that can navigate through the working channel of conventional endoscopes. pCLE includes various mini-probes tailored to esophago-gastric, colonic, and cholangio applications, increasing its versatility across tissues (5). While eCLE offers higher resolution (0.7 μm vs. 1 μm) and adjustable of depth scanning (0–250 μm below surface), pCLE, with its fixed depth of approximately 50 μm, is easier to implement due to its compatibility with conventional endoscopes (2, 6). Despite its lower resolution, it is crucial to emphasize that eCLE has a noteworthy advantage: it does not necessitate endoscopic changes, enabling simultaneous histological evaluation and potential therapeutic intervention during a single endoscopic session. However, eCLE was withdrawn from the market in 2014, and thus, CLE now specifically refers to pCLE in this review.

The utility of CLE is further enhanced by contrast agents, which improve resolution and expand clinical applications (3). Intravenous fluorescein is the most commonly used contrast agent, with a well-established safety profile in over 25 years of use in medical fields, primarily ophthalmology (7). Particularly fluorescein, distributed in the extracellular matrix and cytoplasmic compartment, aids the diagnosis of tumors by selectively staining nuclei, enhancing the visualization of blood vessels and neoplastic angiogenesis, and exploiting vascular leakage (7–9). Among the advantages of fluorescein, its rapid effect is especially noteworthy, with signals detectable 30 s post-administration and optimal image quality within the first 8 min (10). Topical acriflavine, another contrast agent, stains epithelial nuclei and is particularly effective in visualizing neoplastic cells across various conditions (11, 12).

CLE maintains an excellent safety profile. Mild adverse events related to fluorescein, such as nausea, transient hypotension, rash, and mild epigastric pain, occur in 1.4% of patients, with anaphylaxis and other severe events being exceedingly rare (13, 14) Adverse events of acriflavine have not been documented in current literature.

Despite its advantages, CLE use implies certain limitations, including high costs and the need for standardized training (15). However, studies report a short learning curve (16). Commercial CLE systems exceed $8,000, although low-cost alternatives, such as the $5,000 system described by Pierce et al., are emerging (3, 17). These economic and logistic factors should be considered when evaluating CLE's integration into clinical practice.

**Figure 2:**
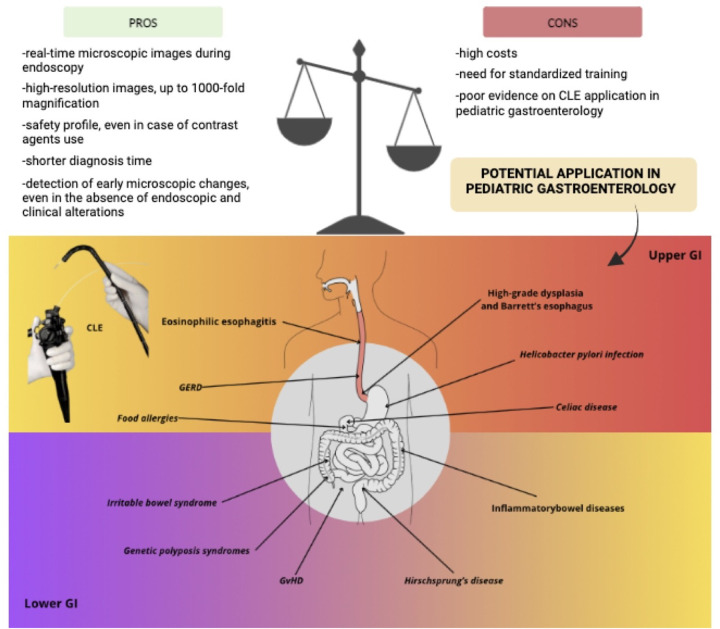
Pros and cons of confocal Laser endomicroscopy and its application in pediatric gastroenterology (figure created with canva - www.canva.com). Current applications are highlighted in **bold** while potential applications are shown in *italics.*

**Figure 3:**
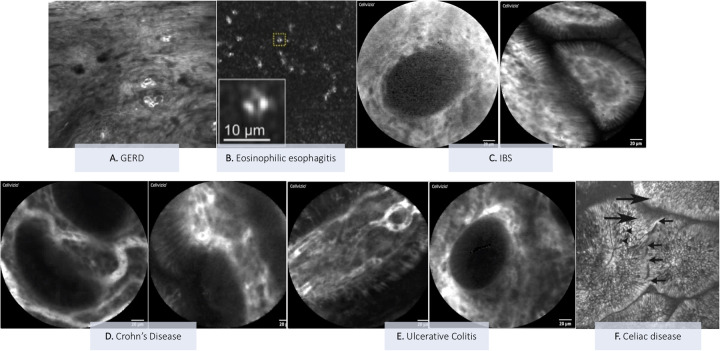
Explanatory pictures of the main applications of confocal Laser endomicroscopy in pediatric gastroenterology. **(A)** GERD—Image form: Venkatesh K, et al. *Gastrointest Endosc* 2012 (20) **(B)** Eosinophilc esophagitis—Image from: Yoo H et al. *Gastrointest Endosc* 2011 (22). **(C)** IBS: duodenum (Figure acquired at the Pediatric Gastroenterology and Liver Unit—University Hospital Umberto I, Sapienza University of Rome, Italy). **(D)** Crohn's disease: mild colonic activity (figures acquired at the Pediatric Gastroenterology and Liver Unit—University Hospital Umberto I, Sapienza University of Rome, Italy). **(E)** Ulcerative colitis: ileum (left image) and colon (right image) (Figures acquired at the Pediatric Gastroenterology and Liver Unit—University Hospital Umberto I, Sapienza University of Rome, Italy). **(F)** Celiac disease: duodenal villi with loss of the cellular architecture (large arrows) of the surface epithelium, decrease in goblet cells (arrowheads) and intervillous bridging (small arrows)—Image from: Venkatesh K et al. *J Pediatr Gastroenterol Nutr* 2010 (28).

**Figure 4:**
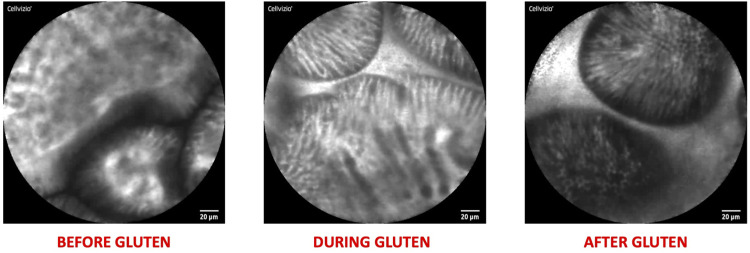
Explanatory pictures of the main applications of confocal Laser endomicroscopy in pediatric gastroenterology—food allergy (figures acquired at the pediatric gastroenterology and liver unit—university hospital umberto I, sapienza university of Rome, Italy).

Figure 2 represents pros and cons of CLE and offers a summary of the current and potential applications in pediatric gastroenterology, which are further discussed in this review. Moreover, some explanatory pictures of the main applications are collected in Figures 3, 4.

## 4 Upper gastrointestinal tract and small bowel

### 4.1 Gastroesophageal reflux disease (GERD)

While the use of CLE in diagnosing high-grade dysplasia and Barrett's esophagus in adults is well-established (18), limited evidence supports its application in benign conditions common in both adults and children, such as GERD.

In adults with suspected GERD, CLE has shown potential, particularly in diagnosing non-erosive reflux disease. Chu et al. demonstrated CLE's high specificity by identifying hallmark features such as dilatation of intercellular spaces and increased intra-papillary capillary loops (19). Conversely, Jong et al. reported significant variability in intra-papillary capillary loops, with larger diameters, numbers and cross-sectional areas observed in erosive reflux disorder compared to non-erosive reflux disease (20). These findings underscore the limitations of CLE as a standalone diagnostic tool, suggesting that it cannot yet replace conventional esophagogastroduodenoscopy (EGD) with biopsy sampling in GERD diagnosis. Microscopic characterization of both erosive and non-erosive forms of GERD requires further refinement before CLE can be widely adopted in clinical practice.

The application of CLE in pediatric GERD has also been explored. Venkatesh et al. identified a potential role for CLE by measuring the surface-to-papillary distance, a parameter reflective of papillary elongation in GERD. This study found significant differences in this measurement between symptomatic and asymptomatic children, correlating CLE findings with histological evaluation (21).

Despite these promising results, further research is needed to investigate additional histological and clinical markers alongside papillary elongation to establish a more comprehensive understanding of CLE's diagnostic capabilities in pediatric GERD. Likely, its use in clinical practice could help better define non-erosive gastroesophageal reflux disease (NERD), saving time and additional exams (e.g., pH-impedance monitoring or BRAVO capsule).

### 4.2 Eosinophilic esophagitis (EoE)

Neumann et al. investigated the potential use of CLE in diagnosing EoE in children. They reported a case of an 18-year-old male presenting with symptoms consistent with EoE who underwent both EGD and CLE with intravenous fluorescein administration. CLE revealed hallmark features such as dilated intracellular spaces, capillary ectasia within the squamous epithelium, contrast agent leakage, and small cells suspicious of eosinophils (22). Further supporting CLE's role in EoE, Yoo et al. demonstrated the accurate count of eosinophils using this technology in pediatric patients with EoE. Their study also highlighted high intra- and interobserver agreement, alongside high sensitivity for detecting other histological features of EoE, such as eosinophilic abscesses, degranulation, and basal cell hyperplasia (23).

The two reported studies primarily focused on EoE diagnosis, leaving open the possibility of utilizing CLE for follow-up evaluations in EoE. Given that children with EoE require frequent endoscopic monitoring with biopsy sampling, CLE might offer a less invasive alternative, potentially reducing the need for biopsies while still providing detailed histological insights.

Furthermore, since esophageal biopsy samples represent only a limited portion of the mucosa, they may not accurately reflect the overall extent of inflammation (24). In this context, CLE could be a valuable tool in EoE management, as it enables simultaneous macroscopic and microscopic evaluation of the entire esophagus.

### 4.3 Helicobacter pylori infection

The application of CLE for diagnosing Helicobacter pylori (Hp) infection holds promises. Ji et al. conducted a study on adults, demonstrating high diagnostic performance of CLE, with a reported accuracy, specificity, and sensitivity rates of 92.8%, 89.2%, and 95.7%, respectively. In this study, the diagnosis was based on identifying specific features, including white spots, neutrophils, and microabscesses (25). To date, no studies have explored the use of CLE for diagnosing *Hp* infection in the pediatric population, where diagnosis relies on culture, molecular tests, and histopathology according to the Sydney system (26). However, it is reasonable to hypothesize that CLE could support these diagnosis procedures by guiding biopsies, thereby improving diagnostic accuracy compared to random biopsy sampling.

### 4.4 Celiac disease

According to the 2020 guidelines issued by the European Society Paediatric Gastroenterology, Hepatology and Nutrition Guidelines (ESPGHAN), the diagnosis of celiac disease can be based solely on compatible serological markers alone, provided that anti-transglutaminase antibody titers are sufficiently elevated (27). However, when serological criteria are not fulfilled, the diagnostic process requires EGD with biopsies and subsequent histological examination. In this context, CLE may serve a dual purpose. First, it can aid in the selection of biopsy sites, enhancing the likelihood of identifying characteristic pathological changes, such as increased intraepithelial lymphocytes and villous atrophy. Supporting this application, Valitutti et al. proposed combining two novel endoscopic techniques, specifically narrow band imaging and water immersion technique, with the goal of enhancing diagnostic accuracy by enabling targeted biopsies of affected tissue (28). Additionally, CLE holds the potential to obviate the need for biopsies altogether by providing real-time diagnostic insights.

Venkatesh et al. conducted a study exploring the utility of CLE in diagnosing Celiac disease in children. The study involved 9 patients with suspected Celiac disease and 10 matched controls, all undergoing EGD and CLE. By comparing confocal images with histological findings, the study reported high sensitivity (100%), specificity (80%), and accuracy (81%), along with a robust inter-observer agreement. While CLE effectively identified features consistent with Marsh 3a/b, such as enlarged villi, loss of cellular architecture, decreased goblet cells, and mucosal damage, is proved less effective at detecting increased intraepithelial lymphocytes (29). This limitation suggests that CLE may be less suited for diagnosing celiac disease that differ from Marsh 3a/b.

In adults, evidence regarding the role of CLE in diagnosing Celiac disease remains limited. Zambelli et al. described the use of CLE in 6 women with dyspepsia, 2 of whom were subsequently diagnosed with celiac disease. Their findings demonstrated correlations between histological results and CLE imaging (30). Similarly, Fort Gasia et al. endorsed the utility of CLE in Celiac disease, reporting high diagnostic accuracy and strong correlation with Marsh grading (31).

In conclusion, although CLE holds promise as a diagnostic tool for Celiac disease, it is not yet a viable substitute for multiple biopsies. However, CLE may serve as an adjunct by guiding biopsy sampling in controversial cases.

## 5 Lower gastrointestinal tract

### 5.1 Inflammatory bowel diseases (IBD)

The potential application of CLE in the and monitoring of IBD has been explored, mainly in adults. Quénéhervé et al. reported that CLE could aid in the diagnosis of IBD by identifying characteristic features such as increased mean inter-crypt distance, wall thickening, and fluorescein leakage (32). A microscopic alteration termed increased epithelial gap density, which reflects intestinal barrier dysfunction, has also been observed in patients with IBD and can be visualized using CLE (33–35). Interestingly, Shavrov et al. applied CLE during colonoscopies in pediatric patients with IBD, detecting increased epithelial gap density in the terminal ileum even in areas with a normal endoscopic appearance. This finding was predictive of disease relapse when observed in more than 3 regions, suggesting that CLE may also have a role in assessing the risk of relapse (35).

Distinguishing between Crohn's disease (CD) and ulcerative colitis (UC) remains a diagnostic challenge but is critical for appropriate disease management. Tontini et al. utilized CLE to identify microscopic alterations of CD and UC, correlating these findings with well-established histopathological features. This study highlighted the potential of CLE for real-time differentiation of IBD types, also by introducing a scoring system, the Endomicroscopy Assessment (IDEA), which provides a quantitative approach to distinguishing CD from UC based on CLE findings (36).

In adults, the application of CLE for assessing CD activity has shown promising results, offering significant advantages over standard endoscopic techniques. Several CLE findings have been associated with active CD, including increased colonic crypt tortuosity, enlarged crypt lumen, microerosions, augmented vascularization, and increased cellular infiltrates within the lamina propria, whereas quiescent CD has been characterized by increased crypt and goblet cell number (37). Several alterations observed using CLE, such as focal cryptitis and cryptal architectural distortion, have been identified as potential risk factors for treatment escalation, suggesting that CLE could play a role in predicting relevant clinical outcomes (38). Furthermore the major axis/minor axis ratio of crypt lumens, as visualized through CLE, has been shown to correlate with active mucosal inflammation on histological assessment in CD (39). Building on these findings, Neumann et al. proposed a Crohn's Disease Endomicroscopic Activity Score (CDEAS) to quantify disease activity based on CLE observations. CDEAS demonstrated a strong correlation with C-reactive protein, a widely recognized marker of inflammation, underscoring its potential as a reliable indicator of CD activity (37).

CLE also shows promise for predicting disease recurrences (40–42). Notably, the recent prospective ERIca trial demonstrated that intestinal barrier healing, as assessed by endomicroscopy, is associated with a reduced risk of disease progression and provides superior predictive value compared to endoscopic and histologic remission (43). Moreover, Kiesslich et al. reported that increased fluorescein leakage, evaluated using the CLE Watson score for intestinal permeability (44), predicts clinical recurrence within 12 month, with a specificity of 91% and a sensitivity of 63% (41). Studies in adult populations also suggest that CLE may predict therapeutic response, particularly to biologic therapies targeting tumor necrosis factor (anti-TNF). Atreya et al. conducted an intriguing study investigating *in vivo* TNF expression using CLE with fluorescent-labeled adalimumab administration. Their findings revealed that a higher number of TNF-expressing cells on CLE correlated with improved short-term response rates at 12 weeks of anti-TNF therapy. Moreover, mucosal healing was observed in 73% of patients with high TNF expression at follow-up intervals of 10 and 24 months (45).

In adults with UC, CLE has been recognized as a valuable tool for assessing disease activity. Maione et al. utilized CLE to identify abnormalities consistent with inflammation, such as fused crypts, in patients with active UC. Additionally, fluorescein leakage observed by CLE was found to correlate with both the Mayo score and histological evidence of inflammation, supporting its utility in providing real-time visualization of microscopic changes associated with UC activity (46).

The current literature highlights several potential applications of CLE in IBD. However, its adoption in clinical practice remains limited, pointing the need of future search on larger cohorts to validate CLE's utility in both the diagnosis and follow-up of IBD. Notably, CLE offers the unique capability to assess disease activity even in areas of macroscopically normal mucosa, a feature that could enhance diagnostic accuracy. Furthermore, the potential of CLE to predict therapeutic responses presents an exciting avenue for exploration. Larger studies, including those focusing on pediatric populations, where treatment options are still limited, could significantly advance the field and establish CLE as integral component of IBD management.

### 5.2 Hirschsprung's disease (HD)

Recent studies have explored the application of CLE for visualizing the enteric nervous system in the diagnosis of HD, a congenital disorder characterized by the absence of ganglion cells in the distal colon, resulting in functional bowel obstruction. The ability of CLE to provide intraoperative visualization of the enteric nervous system could have significant implications for both the diagnosis and management of HD.

An ex vivo study published in 2019 demonstrated that CLE allowed clear visualization of the enteric nervous system in surgically resected intestine segments, with a high concordance rate (88.4%) between confocal images and histopathological examination (47). Additionally, Harada et al. confirmed the technical feasibility of using CLE for *in vivo* visualization of the enteric nervous system during surgery in patients with HD. This real-time imaging capability could facilitate timely diagnostic and therapeutic decisions in the surgical management of HD (48).

The findings underscore the potential utility of CLE in the enhancing the accuracy and efficiency of HD management, enabling rapid and reliable differentiation between aganglionic and ganglionic segments, thereby improving diagnostic precision and appropriate therapeutic interventions.

## 6 Other applications

### 6.1 Irritable bowel syndrome and food intolerances

The application of CLE in functional disorders, such as irritable bowel syndrome (IBS), and in food allergies has been explored in adults. Although IBS is traditionally classified as a functional disorder, CLE has revealed inflammatory microscopic alterations in adults with IBS, such as increased epithelial gap density, increased epithelial leaks, alterations in shape, size and distribution of the crypts and increased capillary density. Notably, these features has been identified even in individuals with macroscopically normal mucosa on conventional endoscopy (49, 50). The observation of micro-inflammation in IBS patients raises intriguing questions about the potential role of anti-inflammatory drugs in managing these conditions. However, further research is required to elucidate the underlying pathogenetic mechanisms and consequently inform optimal therapeutic approaches for IBS.

Moreover, CLE has been explored in patients with symptoms suggestive of IBS and suspected food intolerance. Studies by Fritscher-Ravens et al. identified microscopic anomalies, such as increased intraepithelial lymphocytes, elevated duodenal eosinophils, increased epithelial leaks, and widened intervillous spaces, following food challenges. These findings suggest a potential role for CLE in guiding dietary exclusions by identifying food-specific triggers (51, 52). Consistently, a narrative review by Balsiger et al. highlighted that CLE can reveal acute mucosal alterations after food administration in non-celiac patients without demonstrable allergic sensitization, but who present with IBS-like symptoms upon exposure to a specific foods (53). Collectively, these findings raises the possibility that CLE could aid in reclassifying cases initially diagnosed as IBS but potentially linked to food allergies or sensitivities.

Although the insights provided by these studies are intriguing, it is challenging to envision similar applications in children, where the decision to perform endoscopy is carefully considered based on the potential benefits and risks. As a result, indications for endoscopy in pediatric patients typically fall outside the scope of conditions such as food allergies or IBS.

### 6.2 Genetic polyposis syndromes

Although studies investigating the use of CLE for surveillance in pediatric patients with genetic polyposis syndromes are currently lacking, limited research exists on its application in adults. In one study involving adults with familiar adenomatous polyposis, CLE was employed to characterize duodenal adenomas, including both periampullary and non-ampullary. The primary objective of this approach was to assess the risk associated with the removal of duodenal polyps, aiming to avoid unnecessary excision of non-adenomatous polyps. In this context, CLE demonstrated potential as a tool for decision-making, facilitating informed choices regarding the removal or retention of ampullary or non-ampullary lesions in individuals with familiar adenomatous polyposis (54). Even if the current evidence is limited to adult populations, the application of CLE in pediatric patients with genetic polyposis syndromes, such as familiar adenomatous polyposis, shows promise. CLE's capability for real-time microscopic characterization offers significant advantages for the surveillance of children with these syndromes. By providing immediate microscopic insights during endoscopic procedures, CLE could streamline the diagnostic process, reduce dependence on histopathological analysis, and potentially enable timely interventions within a single endoscopic session.

### 6.3 Graft vs. host disease (GvHD)

Studies in adults have suggested the potential utility of CLE in the diagnosis and management of GvHD, particularly in guiding biopsy sampling. In a pilot study published in 2008 by Bojarski et al. the use of CLE was assessed in patients with acute diarrhea following stem cell transplantation. The study demonstrated high specificity (100%) and sensitivity (74%) for CLE in diagnosing GvHD, emphasizing the CLE's ability to provide rapid, real-time diagnostic insights during endoscopy (55). Subsequent studies in adults have further supported the potential of CLE in diagnosing GvHD (56, 57). Despite the lack of specific studies on CLE in pediatric GvHD, the positive findings in adults point that CLE could also be a valuable diagnostic tool in children.

## 7 Discussion

Although CLE shows considerable promise in this field, its use remains largely investigational at present. In the context of GERD, despite not yet being validated in pediatric population, appears particularly useful. In GERD, it may facilitate the timely identification of NERD, while in EoE it can support the evaluation of eosinophilic infiltration. However, extending these applications to other pediatric gastrointestinal disorders is still challenging, based on current evidence. For celiac disease endomicroscopy can not substitute histologic evaluation. Supporting this, Venkatesh et al. demonstrated that CLE has limitations in detecting non-Marsh 3a/b celiac disease (29). Similarly, while the rose of CLE in pediatric IBD is intriguing, especially regarding the potential value of microscopic alterations such as increased intestinal permeability, its application remain confined to research, and histology continues to be the standard. We also explored the role of CLE in genetic polyposis syndromes. In adults, CLE can provide real-time insights into polyps woth dysplastic or neoplastic features; however, given the rarity of gastrointestinal tumors in children, its clinical applicability in pediatric polyposis syndromes remains limited. Emerging data on CLE for food intolerance and IBS are noteworthy, but given that these conditions seldom justify endoscopic evaluation, CLE in this setting is unlikely to extend beyond the investigational phase in pediatrics. Likewise, the application of CLE to GvHD, *Hp* infection and HD, is supported only by scarse evidence and, at present, is far from routine clinical practice.

Given the paucity of studies on CLE in pediatric gastrointestinale disorders, rigorous research is needed to pave the way for its clinical integration. Establishing dedicated registries and conducting multicenter studies with larger cohorts will be essential to generate high-quality, generalizable evidence. Equally importan is the standardization of image interpretation and the validation of disease-specific variables (i.e., Watson score for intestinal permeability in IBD or the surface to papillary distance in GERD) which could enhance the reproducibility and impact of future studies. Moreover, the advancement of CLE in pediatrics can not be dissociared from the need of structured training. Image acquisition and interpretation demands that pediatric endoscopists undergo tailored educational programs to ensure both accuracy and reliability. We contend that the development of standardized training courses is a fundamental part of research and clinical transtation in this field.

Table 2 summarizes potential applications of CLE in pediatric gastroenterology and proposes disease-specific variables that can be evaluated with CLE, based on current literature.

**Table 2:** Potential applications of confocal laser endomicroscopy (CLE) in pediatric gastrointestinal diseases and disease-specific variables that can be evaluated with CLE.

Disease	Potential applications	Disease-specific variables
GERD^a^	Diagnosis	Surface-to-papillary distance (21), specific features: dilatation of intercellular spaces and increased intra-papillary capillary loops (19)
EoE^b^	Diagnosis and follow-up	Eosinophilic count, specific features: eosinophilic abscesses, degranulation, and basal cell hyperplasia (23)
Hp^c^ infection	Diagnosis	Specific features: white spots, neutrophils, microabscesses (25)
Celiac disease	Diagnosis	Specific features: enlarged villi, loss of cellular architecture, decreased goblet cells, increased intraepithelial lymphocytes (29)
IBD^d^	- Diagnosis and differential diagnosis (Crohns'disease vs. ulcerative colitis) - Assessment of Crohns'disease activity - Assessment of ulcerative colitis activity - Predict disease recurrence	- Increased mean inter-crypt distance, wall thickening, fluorescein leakage, increased epithelial gap density (32–35); use of the scoring system Endomicroscopy Assessment (IDEA) (36) - Specific features for active disease: increased colonic crypt tortuosity, enlarged crypt lumen, microerosions, augmented vascularization, increased cellular infiltrates within the lamina propria (37); major axis/minor axis ratio of crypt lumens (38), use of the Disease Endomicroscopic Activity Score (CDEAS) (37) - Specific features for active disease: fused crypts, fluorescein leakage (46) - Evaluation of the intestinal barrier healing i.e., using the Watson score for intestinal permeability (41)
HD	Diagnosis	Visualization of the enteric nervous system (47, 48)
IBS	Diagnosis	Specific features: increased epithelial gap density, increased epithelial leaks, alterations in shape, size and distribution of the crypts and increased capillary density (49, 50)
Food intolerances	Diagnosis	Specific features following food challenges: increased intraepithelial lymphocytes, elevated duodenal eosinophils, increased epithelial leaks, widened intervillous spaces (51, 52)
Genetic polyposis syndromes	Cancer surveillance	Microscopic alterations suggestive for dysplasia/cancer
GvHD	Diagnosis	

^a^ GERD, gastroesophageal reflux disease; ^b^EoE, eosinophilic esophagitis; ^c^*Hp*, *Helicobacter pylori*; ^d^IBD, inflammatory bowel disease.

## 8 Conclusions

CLE is emerging as a promising tool for diagnosing and monitoring of the major gastrointestinal diseases. Despite challenges in its integration into clinical practice, such as high costs and the need for specialized training, the potential advantages of CLE in digestive endoscopy are noteworthy. By providing real-time, high-resolution microscopic images during endoscopy, CLE offers the potential of a shorter diagnosis time by eliminating the delay typically associated with histopathological examination, offering immediate insights into tissue characteristics and, in some cases, obviating the need for biopsies. In conditions like IBD, CLE may also serve in follow-up care by monitoring disease activity and predicting outcomes. Its ability to detect early microscopic changes, even in the absence of endoscopic and clinical alterations, could enable the prediction of disease recurrence, facilitating timely interventions and potentially improving patient outcome. Additionally, CLE holds promise for cancer surveillance, particularly in patients with genetic polyposis syndromes. However, while current literature offers valuable insights, it remains limited, and future research in children is necessary to provide valuable evidence supporting the integration of CLE into routine clinical practice, particularly in pediatric gastroenterology.
