# Correction: The role of confocal laser endomicroscopy in pediatric gastrointestinal diseases: a narrative review

**DOI:** 10.3389/fped.2025.1716668

**Published:** 2025-10-28

**Authors:** Irene Dalpiaz, Luca Scarallo, Marco Andreini, Sara Renzo, Giusy Russo, Cosimo Ruggiero, Danila Volpe, Paolo Lionetti, Salvatore Oliva

**Affiliations:** ^1^ Gastroenterology and Nutrition Unit, Meyer Children’s Hospital IRCCS, Florence, Italy; ^2^ Department NEUROFARBA, University of Florence, Florence, Italy; ^3^ Pediatric Gastroenterology and Liver Unit, Department of Maternal Infantile and Urological Sciences, Sapienza University of Rome, Rome, Italy

**Keywords:** confocal laser endomicroscopy, pediatric gastrointestinal diseases, inflammatory bowel diseases, food allergy, functional gastrointestinal disorders

Error in figure/table

Wrong content

There was a mistake in Figure 1 as published. The legend does not correspond to the content of the figure. The corrected Figure 1 appears below, in the next page.

**Figure 1:**
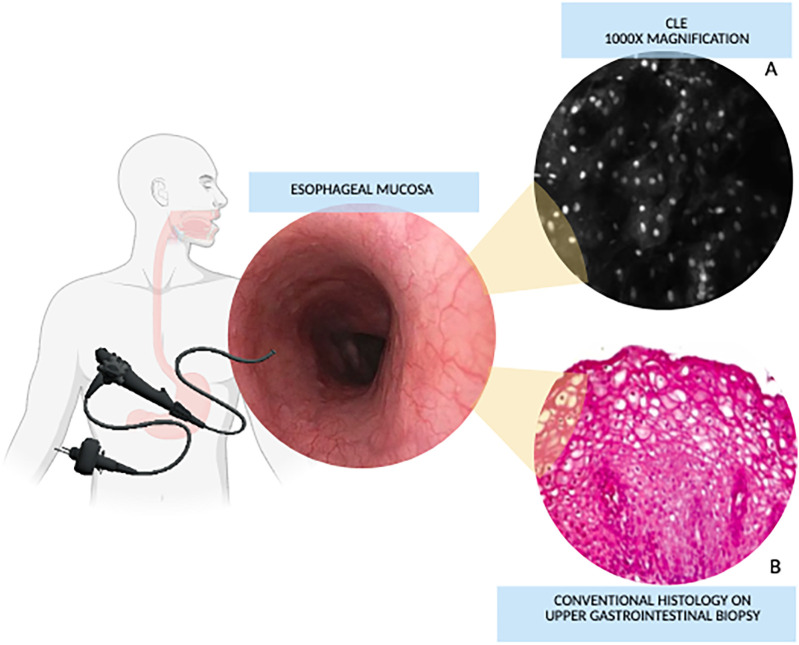
Confocal laser endomicroscopy vs. conventional histology magnification. **(A)** Confocal image of non-keratinized squamous epithelium of the esophagus. **(B)** Histological image of esophagus. Images from: Venkatesh K, et al. Feasibility of confocal endomicroscopy in the diagnosis of pediatric gastrointestinal disorders. *World J Gastroenterol*. (2009) 15:2214–9. doi: 10.3748/wjg.15.2214

